# Supplementary material for: Mitochondrial-cytochrome c oxidase II promotes glutaminolysis to sustain tumor cell survival upon glucose deprivation
Source: Nat Commun. 2025 Jan 2;16:212. doi: 10.1038/s41467-024-55768-9 (PMC11695821; doi:10.1038/s41467-024-55768-9)
Supplement: Supplementary file 1 — Supplementary Information [file 41467_2024_55768_MOESM1_ESM.pdf]

SUPPLEMENTARY INFORMATION

Mitochondrial-cytochrome c oxidase II promotes glutaminolysis to sustain tumor cell survival upon glucose deprivation

Yong Yi[1*], Guoqiang Wang[1], Wenhua Zhang[1], Shuhan Yu[1, 2], Junjie Fei[1], Tingting An[1], Jianqiao Yi[1], Fengtian Li[3], Ting Huang[2], Jian Yang[1], Mengmeng Niu[1], Yang Wang[1*], Chuan Xu[2*], Zhi-Xiong Jim Xiao[1,2,4*]

Affiliations

[1] Center of Growth, Metabolism and Aging, Key Laboratory of Bio-Resource and Eco-Environment, Ministry of Education, College of Life Sciences, Sichuan University, Chengdu, 610064, China.

[2] Department of Oncology & Cancer Institute, Department of Laboratory Medicine and Sichuan Provincial Key Laboratory for Human Disease Gene Study, Sichuan Academy of Medical Sciences, Sichuan Provincial People's Hospital, University of Electronic Science and Technology of China, Chengdu, 610072, China.

[3] School of Biosciences and Technology, Chengdu Medical College, Chengdu, 610500, China.

[4] State Key Laboratory of Biotherapy, West China Hospital, Sichuan University, Chengdu, 610041, China.

*Correspondence:

Zhi-Xiong Jim Xiao: jimzx@scu.edu.cn; Yong Yi: yy-yiyong@scu.edu.cn; Chuan Xu: xuchuan100@163.com; Yang Wang : (wangy90@scu.edu.cn).

Supplementary Figure S1-S12

Supplementary Table 1-3

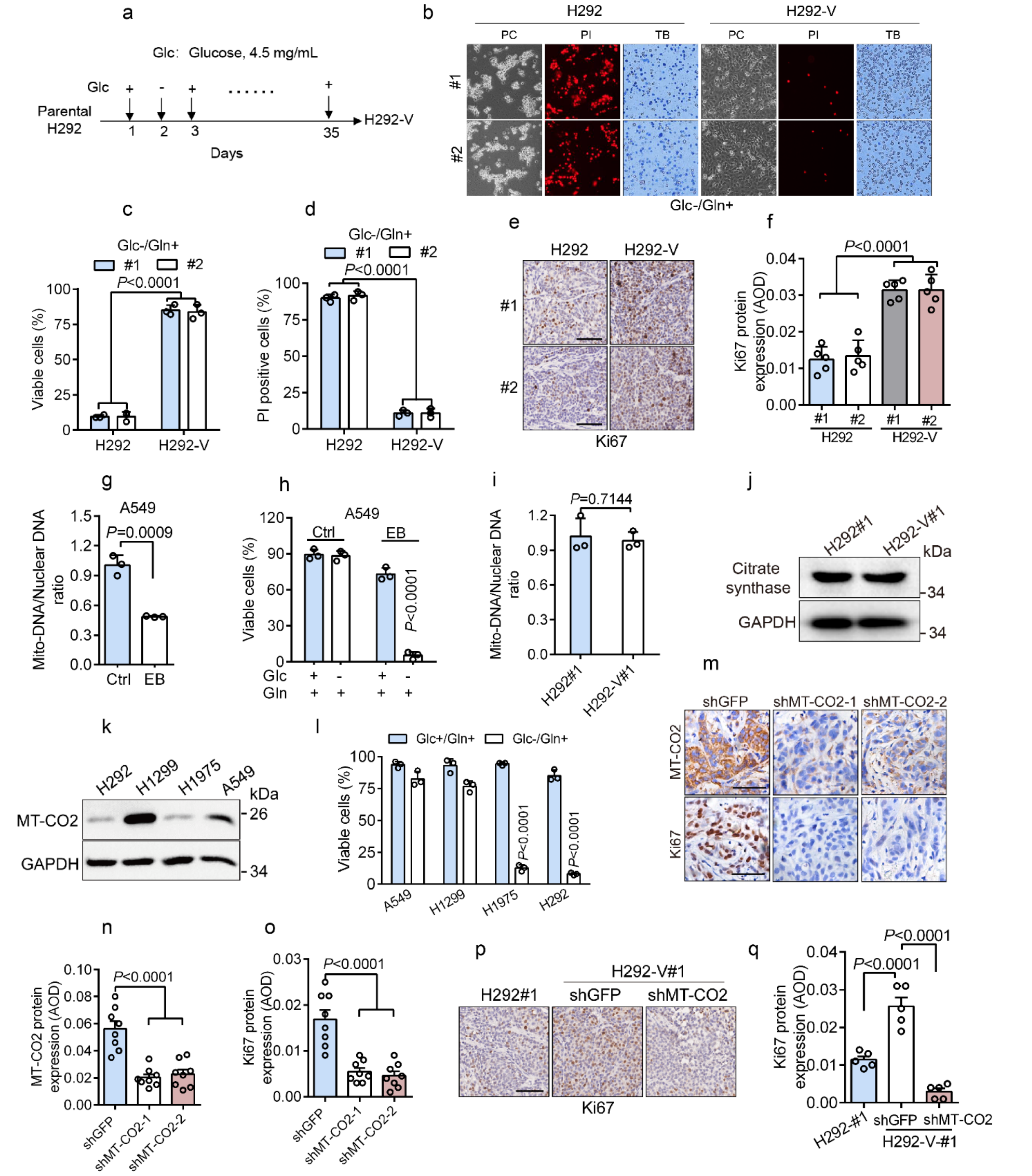

Figure S1. MT-CO2 is critical for tumor cell survival upon glucose deprivation and for tumor growth *in vivo*. (a) An experimental procedure in obtaining sustained viable H292 cells (H292-V) upon glucose deprivation. Two independent screening procedures were performed. **(b-d)** H292 or H292-V cells were grown in DMEM containing 2 mM glutamine in the absence of glucose (Glc-/Gln+) for 48 h. Cell morphologies were recorded by a Phase-contrast (PC) microscope (b). Cell viability was determined by either trypan blue (TB) exclusion assay or propidium iodide (PI) staining assay (c-d, n = 3 independent experiments). **(e-f)** The tumor samples from Figure 1b were subjected to immunohistochemistry staining analyses for Ki67 expression. **(g-h)** A549 cells were treated with or without 300 μM EB (Ethidium bromide) for 7 days. Cells were subjected to qPCR analyses for the ratio of mitochondrial DNA/Nuclear DNA (mtDNA/nDNA) (g, n = 3 independent experiments) or were grown in Glc+/Gln+ or Glc-/Gln+ condition for 24 h followed by trypan blue exclusion

assay for cell viability (h, n = 3 independent experiments). **(i-j)** H292 or H292-V cells were subjected to qPCR analyses for the ratio of mtDNA/nDNA (i, n = 3 independent experiments) or were subjected to western blot analyses (j). The samples derive from the same experiment but different gels for GAPDH and another for citrate synthase were processed in parallel (j). **(k-l)** H292, H1299, H1975, or A549 cells were subjected to western blot analyses (k) or were grown in Glc+/Gln+ or Glc-/Gln+ condition for 24 h, followed by examining cell viability (l, n = 3 independent experiments). **(m-o)** The tumor samples from Figure 1k were subjected to immunohistochemistry staining analyses for MT-CO2 or Ki67 expression (m-o). **(p-q)** The tumor samples from Figure 1q were subjected to immunohistochemistry staining analyses for Ki67 expression. These experiments have been repeated three times with similar results (j-k). Data were presented as mean ± SD (c-d, g-i, l) or SEM (f, n-o, q). Comparisons were performed with one-way ANOVA with Tukey's test (n-o, q) and unpaired two-tailed Student's t-test (c-d, f, g-i, l). Scale bar, 50 μm.

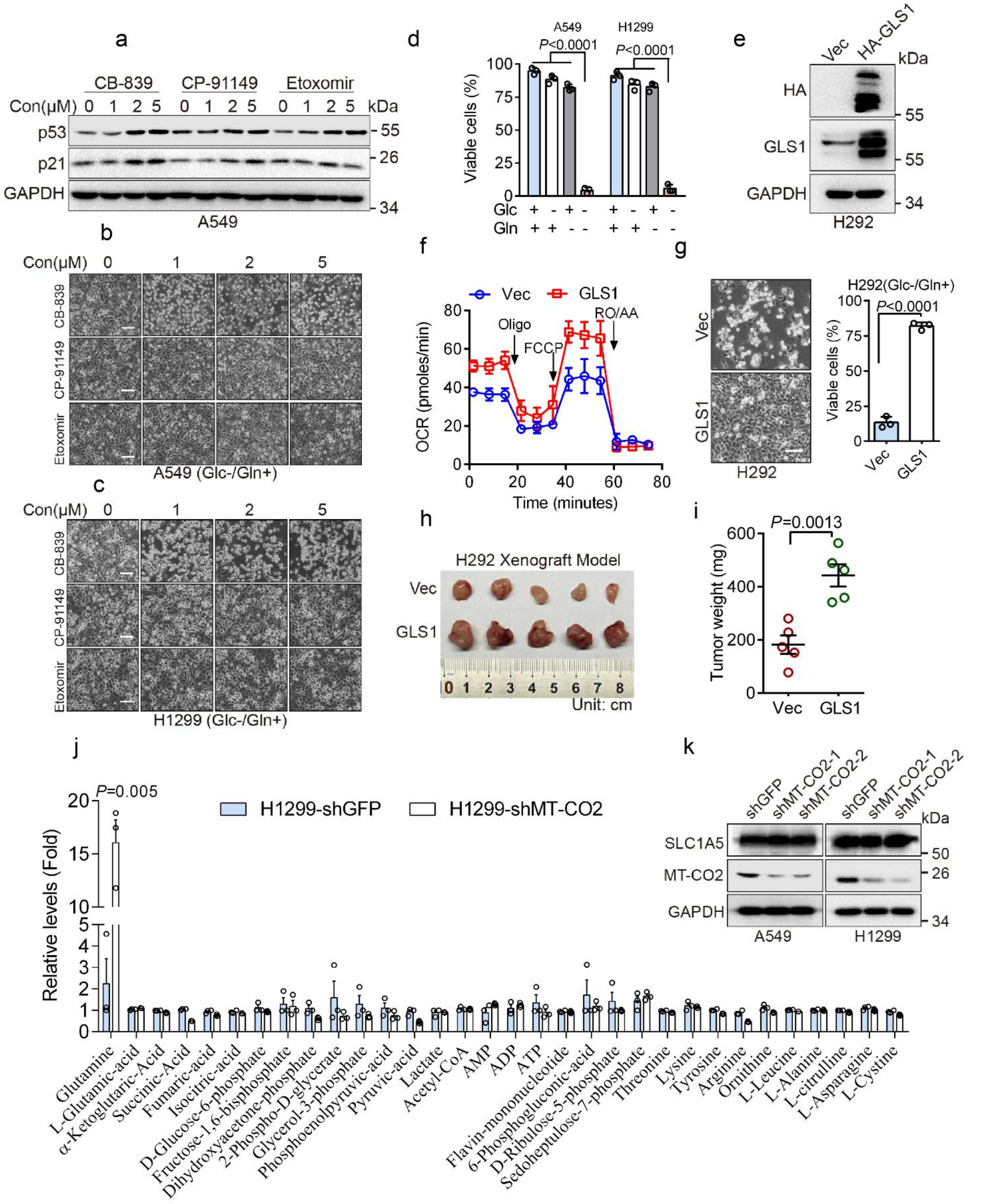

Figure S2. Glutaminolysis is essential for tumor cell survival upon glucose deprivation. (a) A549 cells were treated with an indicated concentration of CB-839 (GLS1 inhibitor), CP-91149 (glycogen phosphorylase inhibitor), or etomoxir (CPT1 inhibitor) for 36 h. Cells were subjected to western blot analyses. **(b-c)** A549 or H1299 cells were treated with an indicated concentration of CB-839, CP-91149, or etomoxir under DMEM containing 2mM glutamine in the absence of glucose (Glc-/Gln+) condition for 36 h (A549) or 48 (H1299). Cell morphologies were recorded by a Phase-contrast microscope. **(d)** A549 or H1299 cells were grown in DMEM containing with or without 2mM glutamine (Gln) in the presence or absence of 4.5 mg/mL glucose (Glc) for 24 h. Cell viability was determined by

trypan blue exclusion assay (n = 3 independent experiments). **(e-f)** H292 cells stably expressing HA-GLS1 were subjected to western blot analyses (e) or were subjected to examine cellular oxygen consumption rate (OCR) (f, n = 3 independent experiments). The samples derive from the same experiment but different gels for GLS1, GAPDH, and another for HA were processed in parallel (e). **(g)** H292 cells expressing HA-GLS1 were grown in Glc-/Gln+ condition for 36 h. Cell viability was determined by either trypan blue exclusion assay (n = 3 independent experiments). **(h-i)** Indicated cells (5×10^5) were subcutaneously inoculated into the right scruff of the female BALB/c nude mouse (n=5/group). Twenty-eight days post-injection, tumors were weighed (i) and photos were taken (h). **(j)** H1299 cells expressing shGFP or shMT-CO2 were subjected to mass spectrometry analyses for energy metabolites as indicated (n=3 biologically independent samples per experiment). **(k)** A549 or H1299 cells expressing shGFP, shMT-CO2-1, or shMT-CO2-2 were subjected to western blot analyses. These experiments have been repeated three times with similar results (a-c, e, k). Data were presented as mean ± SD (d, f-g, j) or SEM (i). Comparisons were performed with one-way ANOVA with Tukey's test (d) and unpaired two-tailed Student's t-test (g, i-j). Scale bar, 50 μm.

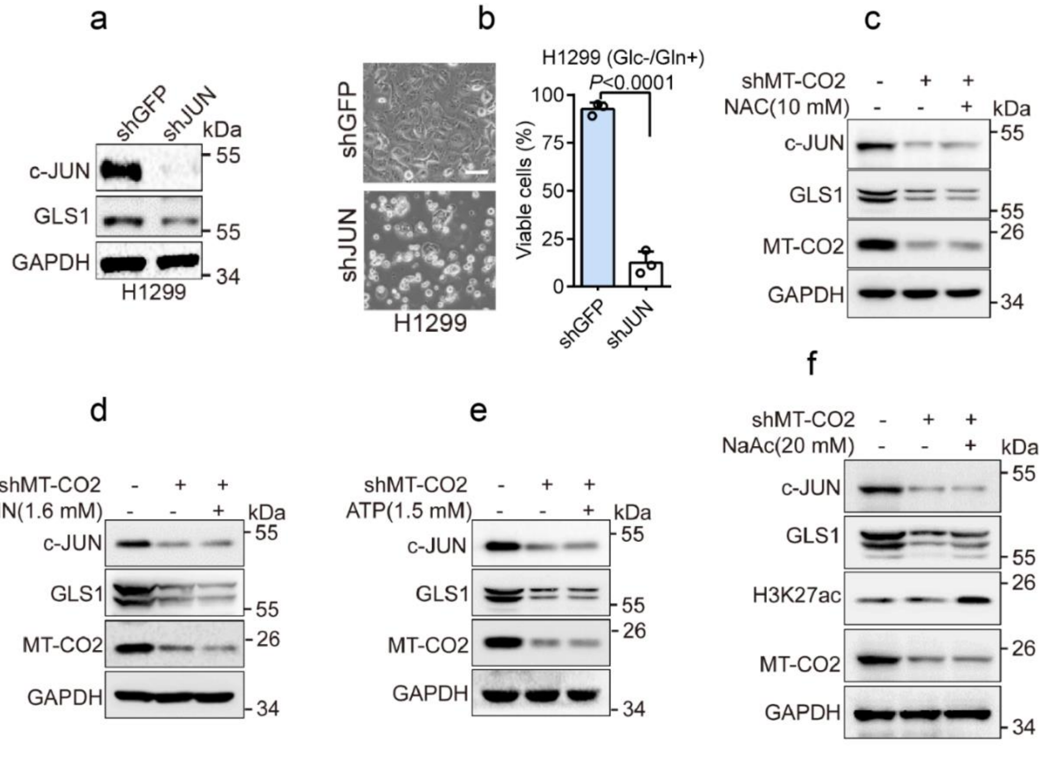

Figure S3. Silencing of c-JUN promotes tumor cell death upon glucose deprivation. (a-b) H1299 cells expressing shGFP or shJUN were subjected to western blot analyses (a) or were grown in DMEM containing 2mM glutamine in the absence of glucose (Glc-/Gln+) condition for 48 h, followed by examining cell viability (b, n = 3 independent experiments). The samples derive from the same experiment but different gels for GLS1, GAPDH, and another for c-JUN were processed in parallel (a). **(c-f)** H1299 cells expressing shMT-CO2 were treated with or without 10 mM N-Acetyl-L-cysteine (NAC, c), 1.6 mM NMN (d), 1.5 mM ATP (e), or 20 mM NaAc (f) for 24 h. Cells were subjected to western blot analyses. (c-e)The samples derive from the same experiment but different gels for GLS1, MT-CO2, GAPDH, and another for c-JUN were processed in parallel. (f) The samples derive from the same experiment but different gels for GLS1, MT-CO2, GAPDH, and another for c-JUN, H3K27ac were processed in parallel. These experiments have been repeated three times with similar

results (a, c-f). Data were presented as mean ± SD (b). Comparisons were performed with unpaired two-tailed Student’s t-test (b). Scale bar, 50 μm.

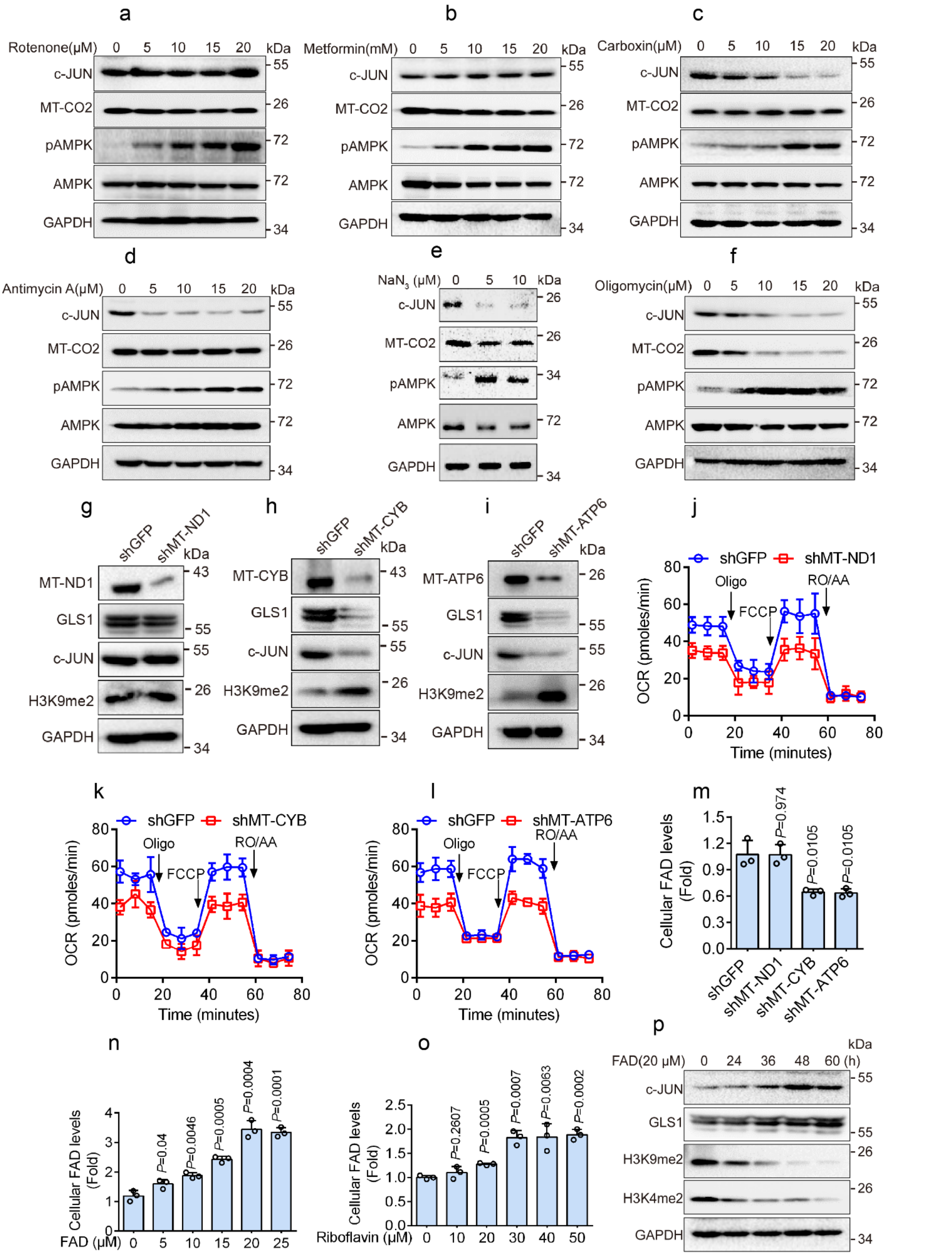

Figure S4. FAD plays a critical role in promoting c-JUN and GLS1 expression. (a-f) Western blot analyses were performed using total cell lysates derived from H1299 cells treated with an indicated respiratory chain complex inhibitor for 24 h, including rotenone

(Complex I inhibitor, a), metformin (Complex I inhibitor, b), carboxin (Complex II inhibitor, c), antimycin A (Complex III inhibitor, d), NaN_3 (Complex IV inhibitor, e) or oligomycin (Complex V inhibitor, f). The samples derive from the same experiment but different gels for MT-CO2, pAMPK, GAPDH, and another for c-JUN, AMPK were processed in parallel (a-f)**. (g-m)** H1299 cells expressing shGFP, shMT-ND1, shMT-CYB, or shMT-ATP6 were subjected to western blot analyses (g-i) or were subjected to examine cellular oxygen consumption rate (OCR) and FAD levels (j-m, n = 3 independent experiments). The samples derive from the same experiment but different gels for MT-ND1, H3K9me2, GAPDH, another for GLS1, and another for c-JUN were processed in parallel (g). The samples derive from the same experiment but different gels for MT-CYB, H3K9me2, GAPDH, another for GLS1, and another for c-JUN were processed in parallel (h). The samples derive from the same experiment but different gels for MT-ATP6, GLS1, GAPDH, and another for H3K9me2, c-JUN were processed in parallel (i). **(n-o)** H1299 cells were treated with an indicated concentration of FAD (n) or riboflavin (o) for 24 h. Cells were subjected to examine cellular FAD levels (n = 3 independent experiments). **(p)** H1299 cells were treated with 20 μM FAD for an indicated time interval. Cells were subjected to western blot analyses. The samples derive from the same experiment but different gels for GLS1, H3K9me2, GAPDH, and another for H3K4me2, c-JUN were processed in parallel (p). These experiments have been repeated three times with similar results (a-i, p). Data were presented as mean ± SD (j-o). Comparisons were performed with unpaired two-tailed Student's t-test (m-o).

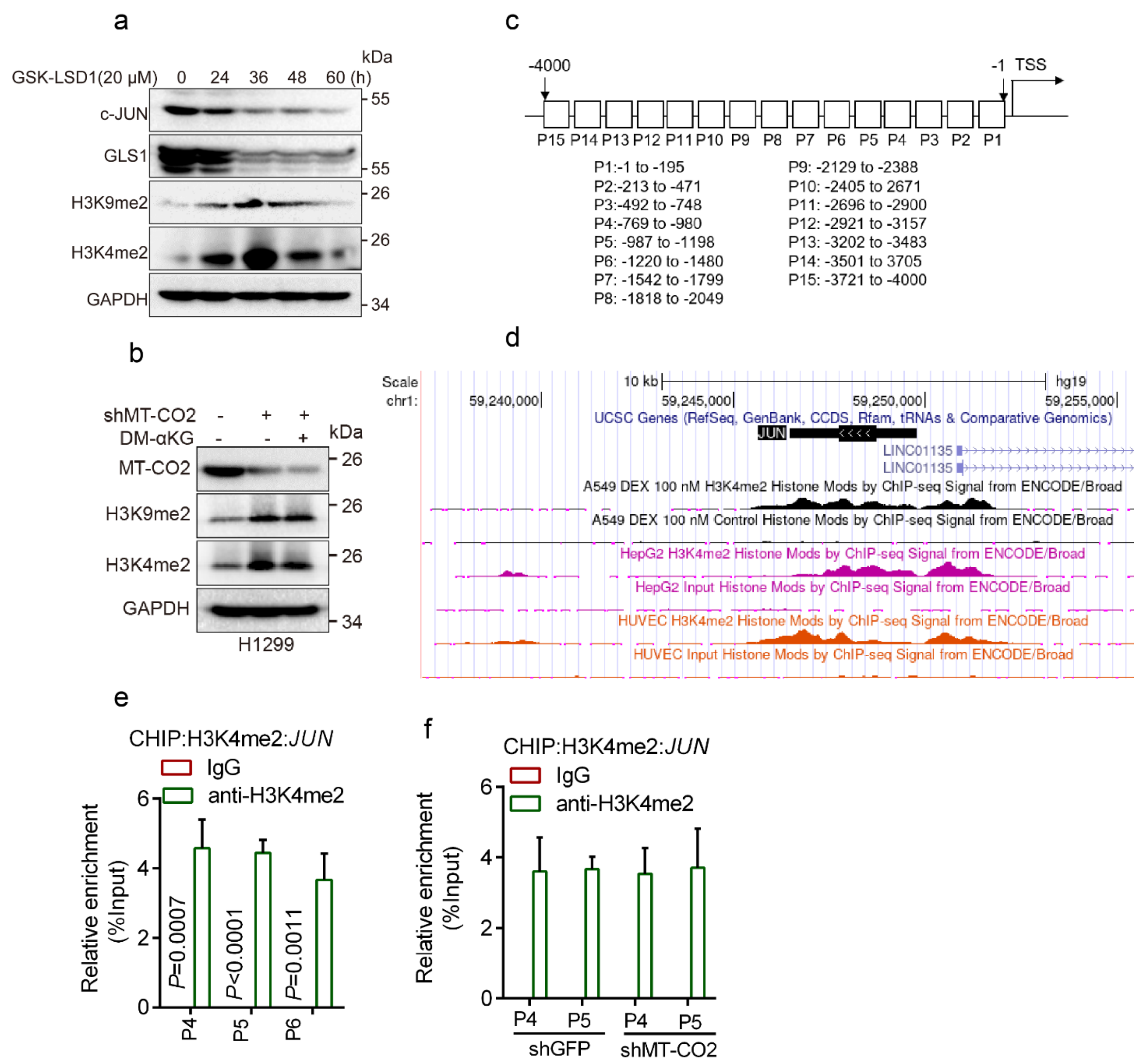

Figure S5. Silencing of MT-CO2 does not affect H3K4me2 binding to the *JUN* gene promoter. (a) H1299 cells were treated with 20 μM GSK-LSD1 for an indicated time interval. Cells were subjected to western blot analyses. The samples derive from the same experiment but different gels for GLS1, H3K9me2, GAPDH, and another for H3K4me2, c-JUN were processed in parallel. **(b)** H1299 cells expressing shMT-CO2 were treated with or without 500 μM dimethyl α-Ketoglutaric acid (DM-αKG) for 48 h. Cells were subjected to western blot analyses. The samples derive from the same experiment but different gels for MT-CO2, GAPDH, another for H3K9me2, and another for H3K4me2 were processed in parallel. **(c)** A schematic presentation depicts potential H3K9me2-binding elements (P1-P15) on the *JUN* gene promoter (-1 to -4000). TSS, transcription start site. **(d)** H3K4me2 could bind to the *JUN* gene promoter region in A549, HepG2, or HUVEC cells. Histone Modifications dataset from ENCODE/Broad Institute was used for this analysis [1]. **(e-f)** H1299 cells were treated with 20 μM GSK-LSD1 for 36 h (e) or H1299 cells were silenced MT-CO2 (f). Cells were subjected to CHIP-qPCR analyses (n = 3 independent experiments). These experiments have been repeated three times with similar results (a-b). Data were presented as mean ± SD (e-f). Comparisons were performed with unpaired two-tailed Student's t-test (e).

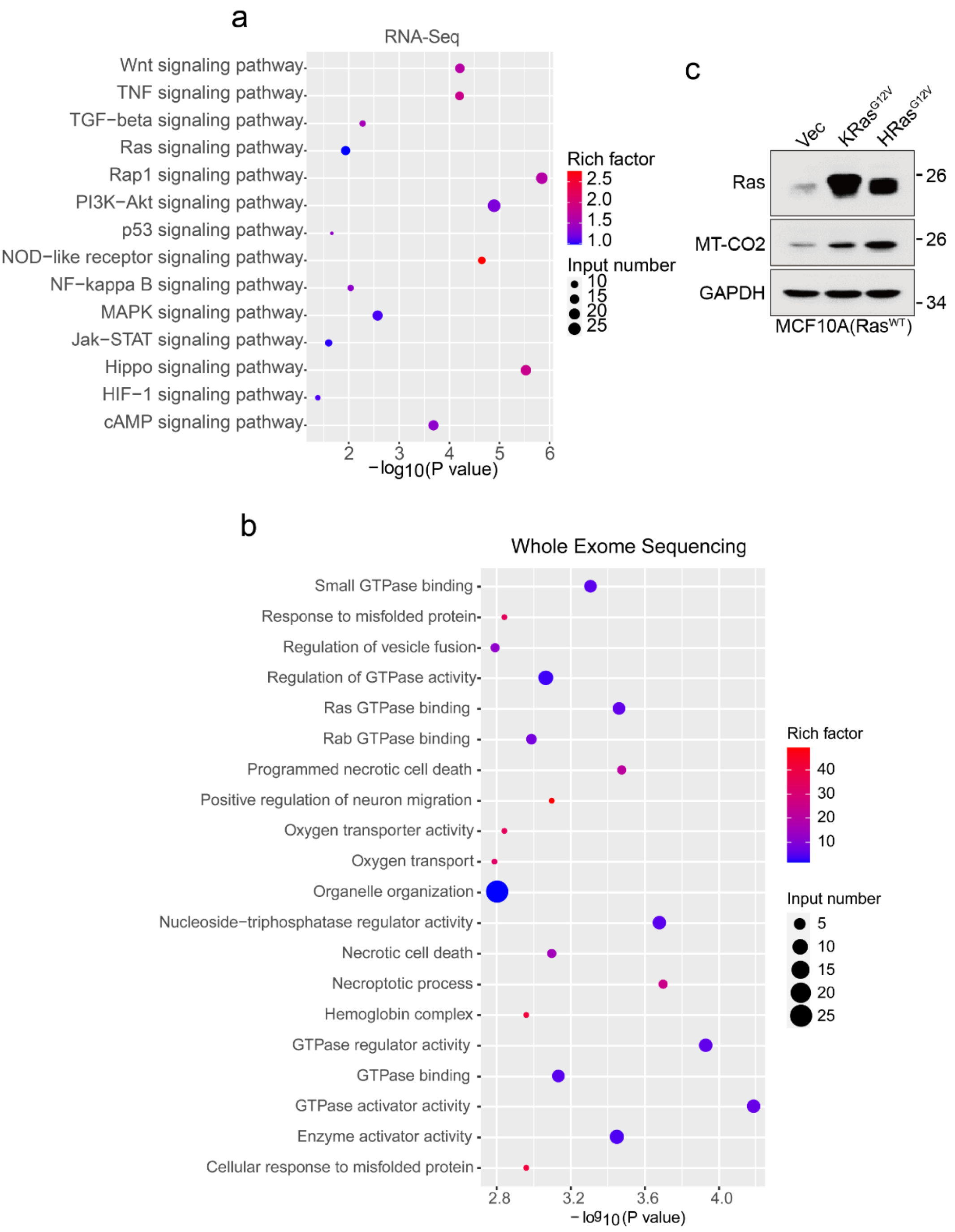

Figure S6. Oncogenic Ras upregulates MT-CO2 expression. (a) H292 or glucose-starvation-resistant H292 (H292-V) cells were subjected to RNA-seq analyses. Differentially expressed genes (DEGs, >2 fold) between H292-V and H292 cells were analyzed. The bubble chart of enrichment KEGG pathways in DEGs was shown (n=2 biologically independent samples per experiment). **(b)** H292 or H292-V cells were subjected to whole exon sequencing analyses. The specific single nucleotide polymorphisms (SNPs) of H292-V cells were subjected to GO enrichment analysis (n=2 biologically independent samples per experiment). **(c)** MCF10A cells stably expressing $KRas^{G12V}$ or $HRas^{G12V}$ were subjected to western blot analyses. This experiment has been repeated three times with similar results. The samples derive from the same experiment but different gels for MT-CO2, GAPDH, and another for Ras were processed in parallel.

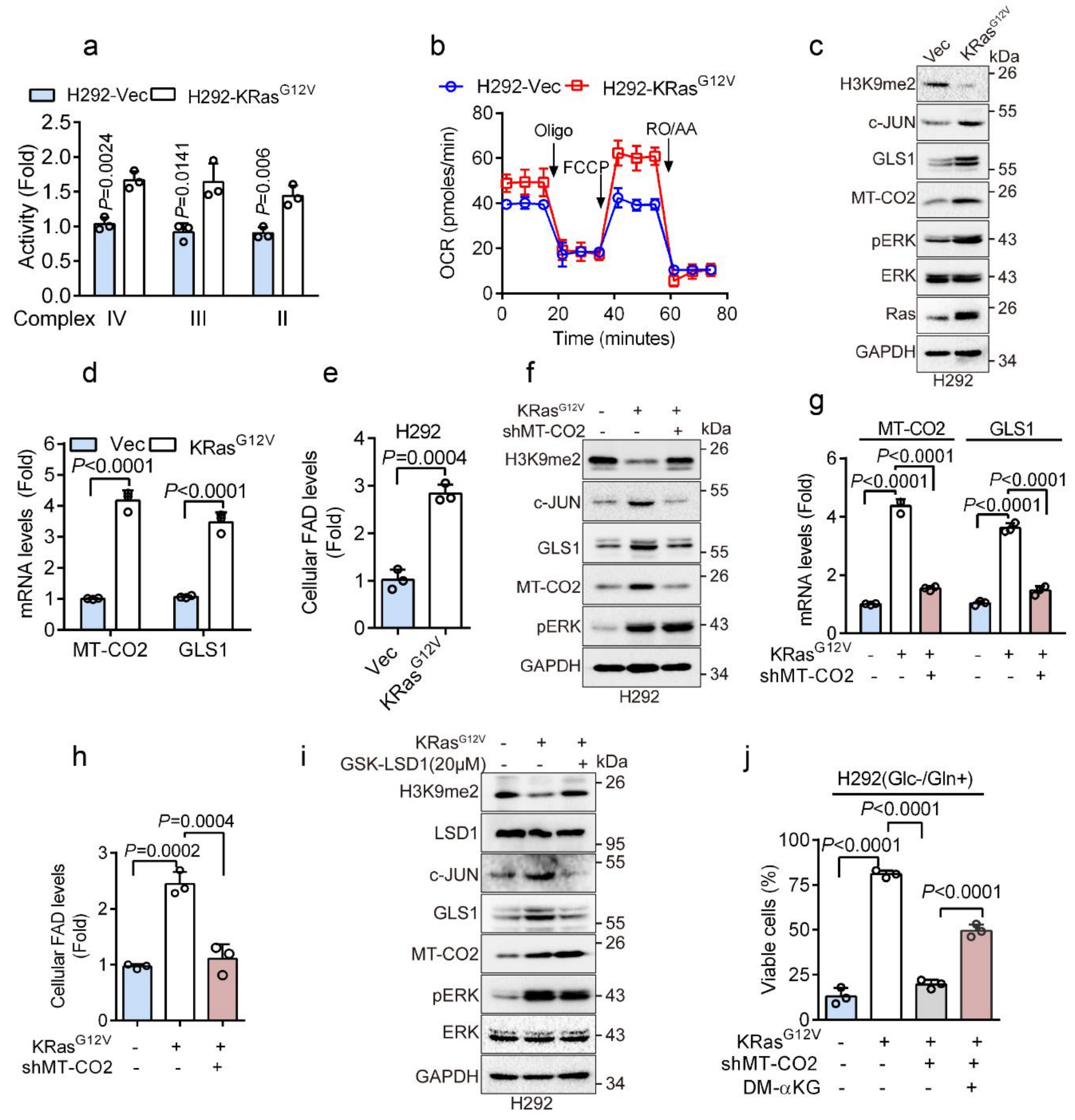

Figure S7. Oncogenic Ras stimulates the MT-CO2-c-JUN-GLS1 axis to promote glutaminolysis and tumor cell survival upon glucose starvation. (a-b) H292 cells expressing KRas^{G12V} were subjected to measurement of the complex (II, III, IV) activity of respiratory chain (a) and oxygen consumption rate (OCR, b) (n = 3 independent experiments). **(c-e)** H292 cells expressing KRas^{G12V} were subjected to western blot (c) or qPCR (d) analyses or were subjected to measurement of FAD levels (e) (n = 3 independent experiments). The samples derive from the same experiment but different gels for MT-CO2, GLS1, GAPDH, another for c-JUN, another for pERK, Ras, and another for ERK, H3K9me2 were processed in parallel (c). **(f-h)** H292-KRas^{G12V} cells expressing shMT-CO2 were subjected to western blot (f) or qPCR (g) analyses or were subjected to examine FAD levels (h, n = 3 independent experiments). The samples derive from the same experiment but different gels for MT-CO2, GLS1, GAPDH, another for c-JUN, H3K9me2 another for pERK were processed in parallel (f). **(i)** H292-KRas^{G12V} cells were treated with or without 20 μM GSK-LSD1 for 36 h. Cells were subjected to western blot analyses. The samples derive from the same experiment but different gels for MT-CO2, GLS1, GAPDH, another for c-JUN, another for pERK, LSD1, and another for ERK, H3K9me2 were processed in parallel. **(j)** H292-KRas^{G12V} cells expressing shMT-CO2 were grown in DMEM containing 2 mM glutamine in the absence of glucose (Glc-/Gln+) with or without 500 μM dimethyl α-

Ketoglutaric acid (DM-αKG) for 24 h. Cell viability was determined by trypan blue exclusion assay (n = 3 independent experiments). These experiments have been repeated three times with similar results (c, f, i). Data were presented as mean ± SD (a-b, d-e, g-h, j). Comparisons were performed with one-way ANOVA with Tukey's test (g-h, j) and unpaired two-tailed Student's t-test (a, d-e).

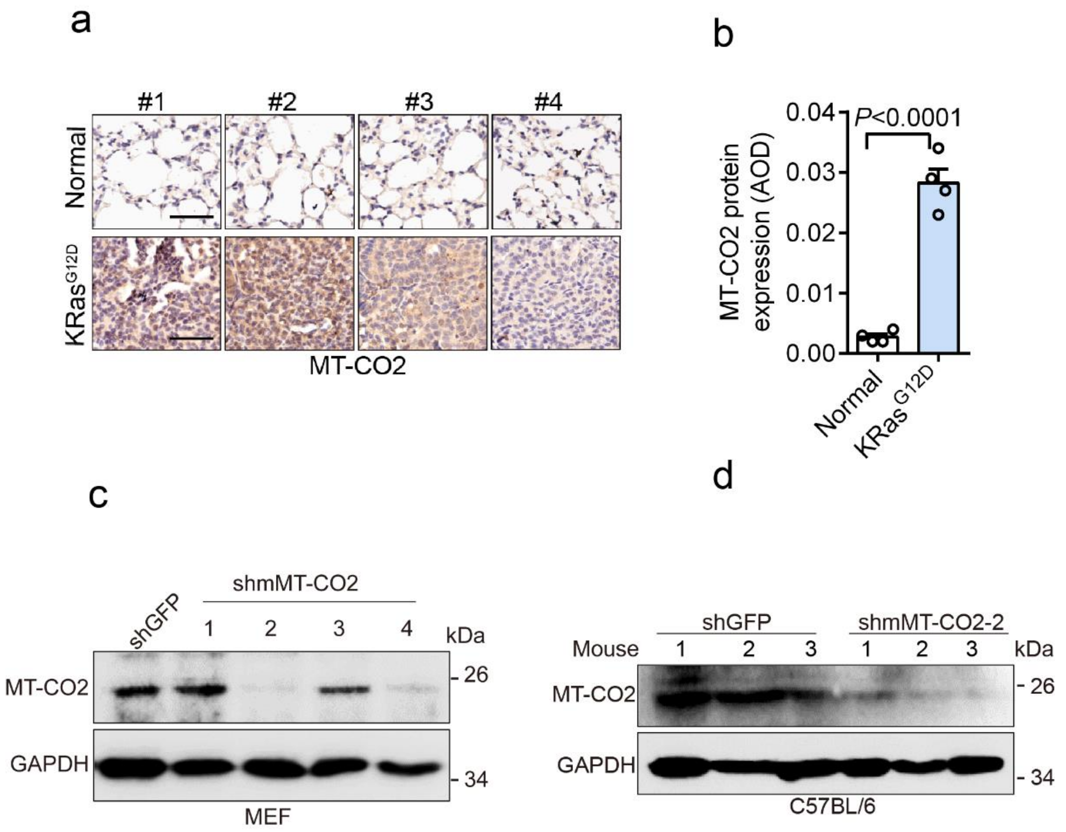

Figure S8. Oncogenic Ras upregulates MT-CO2 expression *in vivo*. (a-b) Normal lung sections derived from C57BL/6 mice (n=4) or lung tumor sections derived from Rosa26-LSL-$KRas^{G12D}$ mice (n=4) were subjected to immunohistochemistry staining for MT-CO2 protein expression (a) with quantitative analyses using average optical density (AOD) (b). **(c)** MEF cells stably expressing shGFP, shmMT-CO2-1, shmMT-CO2-2, shmMT-CO2-3, or shmMT-CO2-4 were subjected to western blot analyses. **(d)** 6-weeks old male C57BL/6 mice (n=3/group) were infected with $2x10^7$ PFU lentivirus-based shGFP or shmMT-CO2 by nasal drip. Seven days after infection, the lungs were excised and subjected to western blot analyses for MT-CO2 protein expression. Data were presented as mean ± SEM (b). Comparisons were performed with unpaired two-tailed Student's t-test (b). Scale bar, 50 μm.

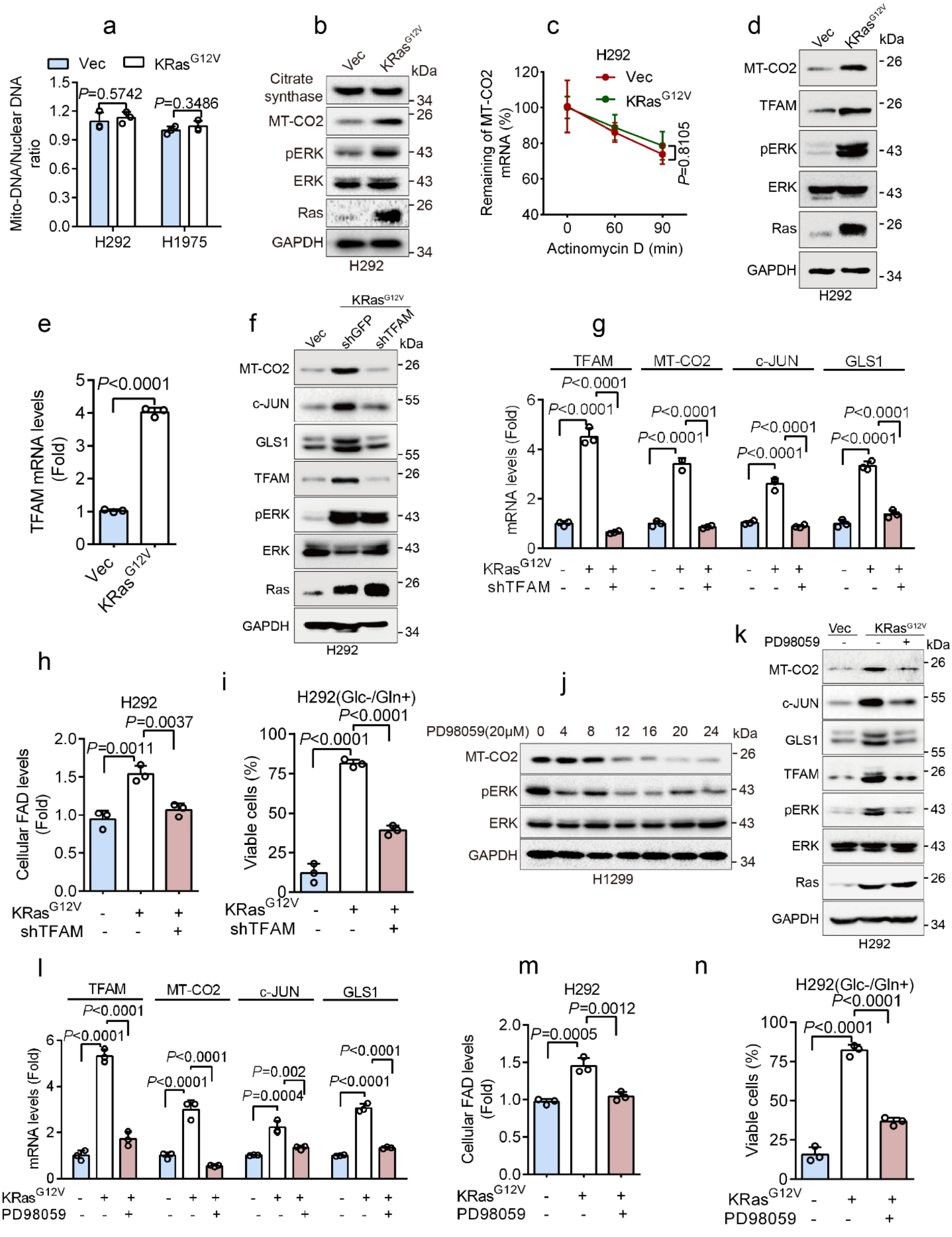

Figure S9. Ras stimulates *MT-CO2* transcription through activation of the ERK-TFAM axis. (a-b) Indicated cells were subjected to qPCR (a, n = 3 independent experiments) or western blot analyses (b). The samples derive from the same experiment but different gels for MT-CO2, GAPDH, another for Ras, pERK, another for citrate synthase, and another for ERK were processed in parallel (b). **(c)** Indicated cells were treated with actinomycin D (10 μM) for an indicated time point, followed by qPCR analyses. The half-life of the MT-CO2 mRNA was shown (n = 3 independent experiments). **(d-e)** Indicated cells were subjected to western blot (d) or qPCR analyses (e, n = 3 independent experiments). The samples derive from the same experiment but different gels for MT-CO2, GAPDH, another for Ras, pERK,

and another for ERK, TFAM were processed in parallel (d). **(f-h)** Indicated cells were subjected to western blot (f) or qPCR (g) analyses or were subjected to examine FAD levels (h) (n = 3 independent experiments). **(i)** Indicated cells were grown in Glc-/Gln+ condition for 24 h, followed by examining cell viability (n = 3 independent experiments). **(j)** H1299 cells were treated with 20 μM PD98059 for an indicated time course, followed by western blot analyses. The samples derive from the same experiment but different gels for MT-CO2, GAPDH, another for pERK, and another for ERK were processed in parallel. **(k-m)** H292-$KRas^{G12V}$ cells were treated with 20 μM PD98059 for 24h, followed by western blot (k) or qPCR(l) analyses or examine cellular FAD levels (m) (n = 3 independent experiments). The samples derive from the same experiment but different gels for MT-CO2, GLS1, GAPDH, another for Ras, pERK, another for c-JUN, TFAM, and another for ERK were processed in parallel (f, k). **(n)** H292-$KRas^{G12V}$ cells were treated with 20 μM PD98059 in Glc-/Gln+ condition for 24 h, followed by examining cell viability (n = 3 independent experiments). These experiments have been repeated three times with similar results (b, d, f, j-k). Data were presented as mean ± SD (a, c, e, g-i, l-n). Comparisons were performed with one-way ANOVA with Tukey's test (g-i, l-n), two-way ANOVA with Bonferroni's test (c), and unpaired two-tailed Student's t-test (a, e).

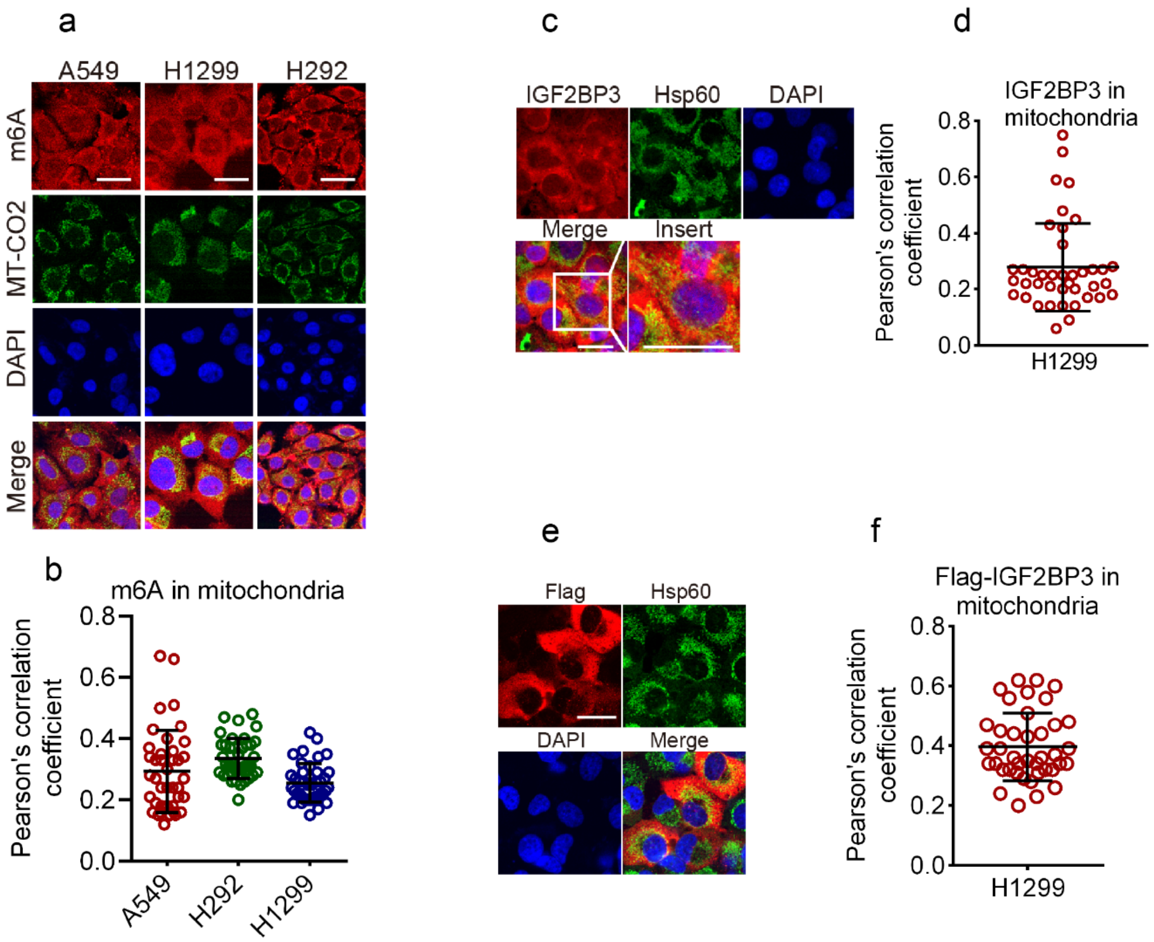

Figure S10. The m6A modification can occur in mitochondria, and IGF2BP3 can localize within mitochondria. (a-b) A549, H1299, or H292 cells were subjected to immunofluorescence staining analyses (a). The co-localization between m6A and mitochondrial marker MT-CO2 (as analyzed by Pearson's correlation coefficient [2,3]) was quantified and statistically analyzed (b). **(c-d)** H1299 cells were subjected to immunofluorescence staining analyses (c). The co-localization between IGF2BP3 and mitochondrial marker Hsp60 was quantified and statistically analyzed (d). **(e-f)** H1299 cells

expressing Flag-IGF2BP3 were subjected to immunofluorescence staining analyses (e). The co-localization between Flag and Hsp60 was quantified and statistically analyzed (f). The Pearson's correlation coefficient was used for the co-localization quantification (b, d, f). 40 cells derived from three independent experiments were randomly chosen and subjected to quantification analyses. Data were presented as mean ± SD (b, d, f). Scale bar, 25 μm.

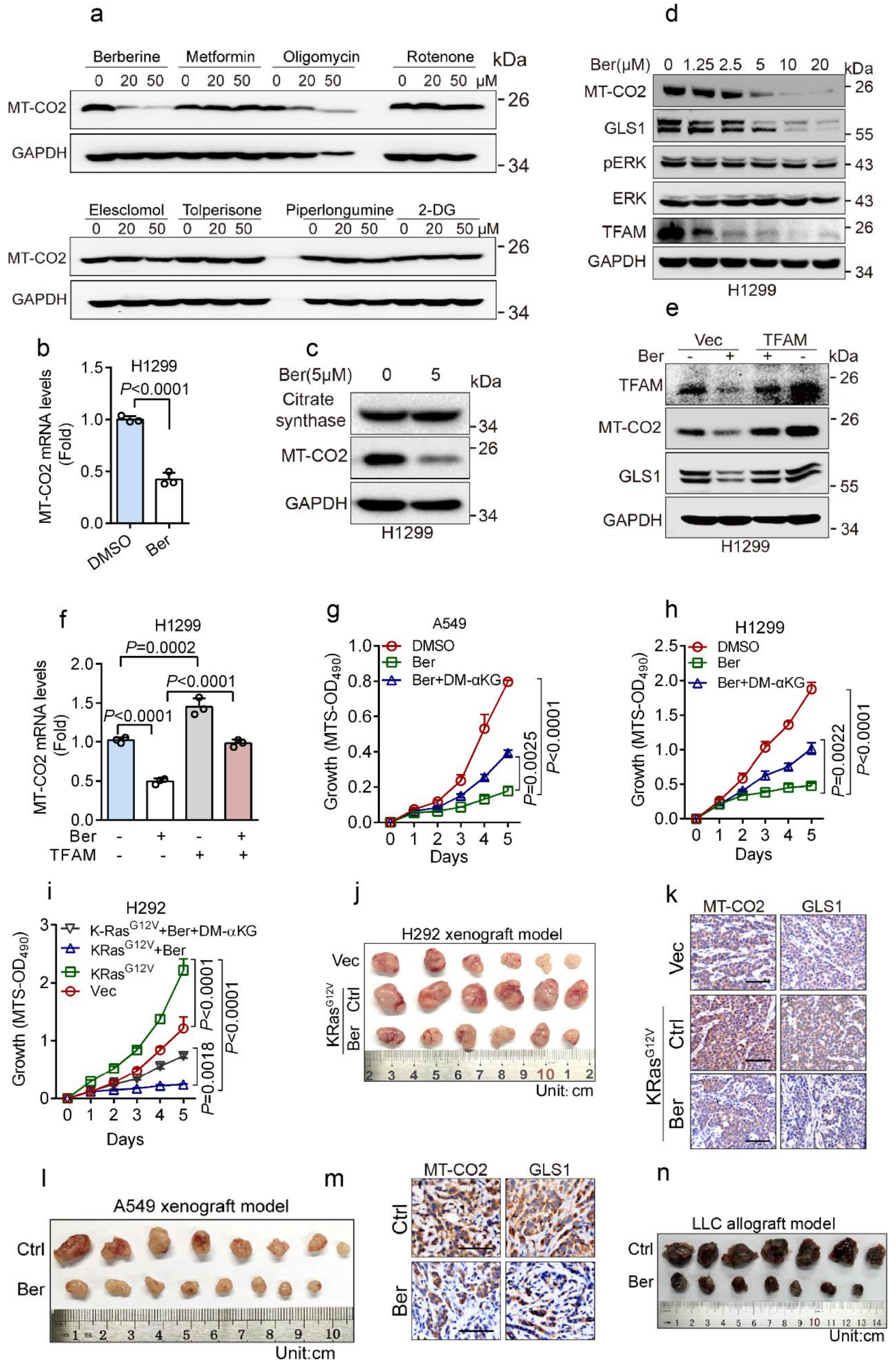

Figure S11. Berberine inhibits MT-CO2 expression via suppressing TFAM to suppress glutamine utilization and tumor growth. (a) A549 cells were treated with an indicated metabolic inhibitor for 24 h prior to western blot analyses. **(b-c)** H1299 cells were treated with or without berberine (5 μM, and thereafter) for 24 h. Cells were subjected to qPCR (b, n = 3 independent experiments) or western blot analyses (c). The samples derive from the same experiment but different gels for MT-CO2, GAPDH, and another for citrate synthase were processed in parallel (c). **(d)** H1299 cells were treated with berberine at an indicated dose for 24 h prior to western blot analyses. The samples derive from the same experiment but different gels for MT-CO2, GLS1, GAPDH, another for pERK, TFAM, and another for ERK were processed in parallel. **(e-f)** H1299 cells expressing TFAM or vector control (Vec) were treated with berberine for 24 h. Cells were subjected to western blot (e) or qPCR (f, n = 3 independent experiments) analyses. The samples derive from the same experiment but different gels for MT-CO2, GLS1, GAPDH, and another for TFAM were processed in parallel (e). **(g-h)** A549 (g) or H1299 (h) cells were treated with berberine in the presence or absence of 500 μM α-Ketoglutaric acid (DM-αKG) for an indicated time interval. Cell growth was determined by MTS analyses (n = 3 independent experiments). **(i)** H292-KRas^{G12V} cells were treated with berberine in the presence or absence of 500 μM DM-αKG for an indicated time interval. Cell growth was determined by MTS analyses (n = 3 independent experiments). **(j-m)** Tumor photos derived from Fig. 7f and 7j were taken (j, l). The tumor samples were subjected to immunohistochemistry staining analyses for MT-CO2 and GLS1 expression (k, m). scale bar, 50 μm. **(n)** Tumor photos derived from Fig. 7n were taken. These experiments have been repeated three times with similar results (a, c, d-e). Data were presented as mean ± SD (b, f-i). Comparisons were performed with one-way ANOVA with Tukey's test (f), two-way ANOVA with Tukey's test (g-i), and unpaired two-tailed Student's t-test (b).

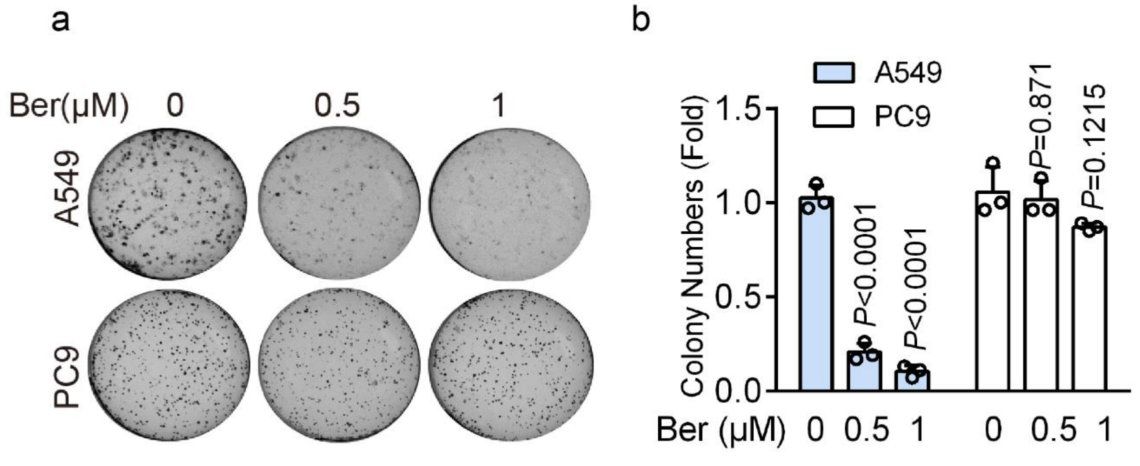

Figure S12. Tumor cells harboring mutant *RAS* alleles are more sensitive to berberine than tumor cells harboring wild-type *RAS* alleles. (a-b) A549 ((KRas^{G12S}) or PC9 (Ras^{WT}) cells were treated with an indicated concentration of berberine for 10 days. Cells were subjected to colony formation analyses (n = 3 independent experiments). Data were presented as mean ± SD (b). Comparisons were performed with one-way ANOVA with Tukey's test (b).

Supplementary Table 1. H292 or glucose-starvation-resistant H292 (H292-V) cells were subjected to whole exon sequencing analyses. The Ras pathway-related gene mutations were shown.

Gene name	H292 mutation	H292-V mutation
KRAS	G483A:R161R	G483A:R161R
HRAS	T81C:H27H	T81C:H27H
NRAS	/	/
ARAF	C624G:A208A, C615G:A205A	C624G:A208A, C615G:A205A
BRAF	A1929G:G643G	A1929G:G643G
CRAF	A1755G:V585V	A1755G:V585V
MEK1	/	/
MEK2	C660A:I220I	C660A:I220I
ERK1	/	/
ERK2	/	/

Supplementary Table 2. Putative m6A site on 13 mitochondrial mRNAs. SRAMP database was used for this analysis (http://www.cuilab.cn/sramp).

mRNA	Position	Sequence	Score	Confidence
MT-CYB	144	CCACAGGACTATTCCTAG	0.646	High
MT-ND5	1083	ATAGGAGGACTACTCAAA	0.636	High
MT-CO2	345	ACTACGGCGGACTAATCTTC	0.612	High
MT-ND5	933	TCAACTAGGACTCATAATA	0.612	High
MT-ND5	1491	TTCCTAGGACTTCTAACAG	0.593	Moderate
MT-ND4	1041	CTCAAGGACTTCAAACTCT	0.591	Moderate
MT-ND1	618	GAGTCCGAACTAGTCTCAG	0.591	Moderate
MT-CO1	1325	CGTTACTCGGACTACCCCGATG	0.581	Moderate
MT-CO1	18	ACCGTTGACTATTCTCTAC	0.577	Moderate
MT-CO1	1482	CCTGGAGTGACTATATGGATGC	0.576	Moderate
MT-ND4	633	CTTTACGGACTCCACTTAT	0.569	Moderate
MT-ND4	1191	ACTTACAGGACTCAACATA	0.564	Moderate
MT-CYB	1053	ATCATTGGACAAGTAGC	0.561	Moderate
MT-CO1	41	AAACCACAAAGACATTGGAACA	0.561	Moderate
MT-ND1	759	TCCCCTGAACTCTACACA	0.557	Low
MT-ND2	246	CTCCGGACAATGAACCATAAC	0.549	Low
MT-CO2	196	CCGTCTGAACTATCCTGC	0.546	Low
MT-ATP6	258	TCCTCGGACTCCTGCCT	0.542	Low
MT-ND5	194	TATCTCGAACTGACACTGA	0.541	Low
MT-CO3	615	GCTTCCACGGACTTCACGTCA	0.54	Low
MT-ND3	335	AAAGGATTAGACTGAACCGAAT	0.539	Low
MT-CYB	1020	TCGGAGGACAACCAGTA	0.53	Low
MT-ATP6	144	AACAATGACTAATCAAAC	0.53	Low
MT-ATP8		/		
MT-ND6		/		
MT-ND4L		/		

Supplementary Table 3. Primers were used in this study.

Target	Application	Sequence
GFP	shRNA	GAAGCAGCACGACTTCTTC
Human MT-CO2	shRNA-1	GAGCAAACCACAGTTTCATGC
Human MT-CO2	shRNA-2	GAGTAGTACTCCCGATTGAAG
Human TFAM	shRNA	CGTGAGTATATTGATCCAGAA
Human c-Myc	shRNA	CAGTTGAAACACAAACTTGAA
Human c-JUN	shRNA	ACAAGTTTCGGGGCCGCAAC
Human IGF2BP3	shRNA-1	GCAGGAATTGACGCTGTATAA
Human IGF2BP3	shRNA-2	CCTCATTCTTATTTCAAGAT
Human KRas	shRNA-1	GACGAATATGATCCAACAATA
Human KRas	shRNA-2	GAGGGCTTTCTTTGTGTATTT
Human MT-ND1	shRNA-1	CTTCTAACCTCCCTGTTCTTA
Human MT-ND1	shRNA-2	AAACAATCTCATATGAAGTCA
Mouse MT-CO2	shRNA-1	AAGCTACGAATATACTGACTA
Mouse MT-CO2	shRNA-2	AAGCACAATAGATGCACAAGA
Mouse MT-CO2	shRNA-3	AACCATAGGGCACCAATGATA
Mouse MT-CO2	shRNA-4	AAGCACAATAGATGCACAAGA
MT-CO2 F	qPCR	CACATGCAGCGCAAGTAGGTCTA
MT-CO2 R	qPCR	GGCAGGATAGTTCAGACGGTTTC
MT-CO1 F	qPCR	CGCCACACTCCACGGAAGCA

MT-CO1 R	qPCR	CGGGGCATTCCGGATAGGCC
MT-CO3 F	qPCR	CACTGGCCCCCAACAGGCAT
MT-CO3 R	qPCR	AGTATCAGGCGGCGGCTTCGA
MT-ND1 F	qPCR	ACCCCCTTCGACCTTGCCGA
MT-ND1 R	qPCR	GGGCCTGCGGCGTATTCGAT
MT-ND2 F	qPCR	GCCCCCGCTAACCGGCTTTT
MT-ND2 R	qPCR	GGGCGATGAGTGTGGGGAGGA
MT-ND3 F	qPCR	TTACGAGTGCGGCTTCGACC
MT-ND3 R	qPCR	ACTCATAGGCCAGACTTAGG
MT-ND4 F	qPCR	CTAGGCTCACTAAACATTCTA
MT-ND4 R	qPCR	CCTAGTTTTAAGAGTACTGCG
MT-ND5 F	Q-PCR	TCGAATAATTCTTCTCACCC
MT-ND5 R	qPCR	TAGTAATGAGAAATCCTGCG
MT-ND4L F	qPCR	TAGTATATCGCTCACACCTC
MT-ND4L R	qPCR	GTAGTCTAGGCCATATGTG
MT-CYB F	qPCR	TCCTACACATCGGGCGAGGCC
MT-CYB R	qPCR	GGTGATTCCTAGGGGGTTGT
MT-ATP6 F	qPCR	CCCTGGCCGTACGCCTAACC
MT-ATP6 R	qPCR	GCTAGGGTGGCGCTTCCAAT
MT-ATP8 F	qPCR	ATGCCCCAACTAAATACT
MT-ATP8 R	qPCR	TTGTGGGGGCAATGAATG

CYTB (DNA) F	qPCR	CCTCATCCTAGCAATAATCCCCATCCT CCATATAT
CYTB (DNA) R	qPCR	ACTTGTCCAA TGATGG TAAAAGG
H2B (DNA) F	qPCR	TGCTGT CTCCATG TTTGAT GTATCT
H2B (DNA) R	qPCR	TCTCTGCT CCCCACC TCTAAGT
GLS1 F	qPCR	CCGGTCGCGGCAATCCTAGC
GLS1 R	qPCR	GTCTGTGGTGGGGCGGTGAG
GLUD1 F	qPCR	CTCCAGACATGAGCACAGGTGA
GLUD1 R	qPCR	CCAGTAGCAGAGATGCGTCCAT
c-JUN F	qPCR	GTACCTGATGAACCTGATGC
c-JUN R	qPCR	GGTCACAGCACATGCCACTT
TFAM F	qPCR	ATGGCGTTTCTCCGAAGCAT
TFAM R	qPCR	CAGATGAAAACCACCTCGGTAA
GAPDH F	qPCR	GGGGAGCCAAAAGGGTCATCATCT
GAPDH R	qPCR	GAGGGGCCATCCACAGTCTTCT
P1 F	CHIP-qPCR	CAGCGGAGCATTACCTCA
P1 R	CHIP-qPCR	CTTATCCAGCCCGAGCTCAA
P2 F	CHIP-qPCR	CAAAACAACTGGCCAGGTTC
P2 R	CHIP-qPCR	CCTTCTTTCTCCTACCCTCC
P3 F	CHIP-qPCR	CTGAACTTGACCGAGATGCA
P3 R	CHIP-qPCR	GGTGGTTGTTGTTTCCCCAC

P4 F	CHIP-qPCR	CGAGTTTCGGATCGCCTAC
P4 R	CHIP-qPCR	GTCTGGCTGCTCGTAGAAG
P5 F	CHIP-qPCR	GCAGAGATTTGAAGTCCAGG
P5 R	CHIP-qPCR	CCCGGTGTTAGTCTACTCC
P6 F	CHIP-qPCR	TAACTCCTGGCAGCCCAGT
P6 R	CHIP-qPCR	GCATATCCTGGGTAGCCTC
P7 F	CHIP-qPCR	GCAATGAACCCAAGGCTG
P7 R	CHIP-qPCR	TGCCTCGCAGTTTCCATC
P8 F	CHIP-qPCR	TCAGGGCAACAGACAGAGG
P8 R	CHIP-qPCR	TGCGGCTTTGAGTGTTCAG
P9 F	CHIP-qPCR	CCACAAGCGCTATTTCCTC
P9 R	CHIP-qPCR	AACACTTATTATCTGCCAGGC
P10 F	CHIP-qPCR	AGGCATAGGAATCGGAACTC
P10 R	CHIP-qPCR	TTGCCTGATCCTCTGACTG
P11 F	CHIP-qPCR	AACACGAGGCCTCCTTCA
P11 R	CHIP-qPCR	AACTCCTTCTCTCTCCAGC
P12 F	CHIP-qPCR	ACCAGGGGAAGGAGAGTAG
P12 R	CHIP-qPCR	TCCTGACTGTGGGGAAGGA
P13 F	CHIP-qPCR	GGAGAGCAGGAAAGCAAAGA
P13 R	CHIP-qPCR	CATCCAGTCACAGCACCAAG
P14 F	CHIP-qPCR	CTCCAACCAGGGCAACAAGA

P14 R	CHIP-qPCR	GTCTCTCTTTTGCCGTCTCAC
P15 F	CHIP-qPCR	CTTTTAGCAAGAAAGGACCTGG
P15 R	CHIP-qPCR	TCGGCTCACTGCAACCTCTA

Reference

1 Ram, O. *et al.* Combinatorial patterning of chromatin regulators uncovered by genome-wide location analysis in human cells. *Cell* **147**, 1628-1639, doi:10.1016/j.cell.2011.09.057 (2011).

2 Vaparanta, K. *et al.* An extracellular receptor tyrosine kinase motif orchestrating intracellular STAT activation. *Nat Commun* **13**, 6953, doi:10.1038/s41467-022-34539-4 (2022).

3 Yan, G. *et al.* Genome-wide CRISPR screens identify ILF3 as a mediator of mTORC1-dependent amino acid sensing. *Nature cell biology* **25**, 754-764, doi:10.1038/s41556-023-01123-x (2023).
