# Supplementary material for: Mitochondrial-cytochrome c oxidase II promotes glutaminolysis to sustain tumor cell survival upon glucose deprivation
Source: Nat Commun. 2025 Jan 2;16:212. doi: 10.1038/s41467-024-55768-9 (PMC11695821; doi:10.1038/s41467-024-55768-9)
Supplement: Supplementary file 2 — Description of Supplementary Information [file 41467_2024_55768_MOESM2_ESM.docx]

**File Name: Supplementary Data 1**

Description: Analyses of the MT-CO2 effects on mRNA expression by RNA sequencing in A549 cells.

**File Name: Supplementary Data 2**

Description: Analyses of the gene mutations by whole exon sequencing in parental H292 cells.

**File Name: Supplementary Data 3**

Description: Analyses of the gene mutations by whole exon sequencing in glucose-starvation-resistant H292 cells (H292-V).

**File Name: Supplementary Data 4**

Description: Analyses of the mRNA expression by RNA sequencing in glucose-starvation-resistant H292 cells (H292-V) or parental H292 cells.

**File Name: Supplementary Data 5**

Description: Examine the effects of MT-CO2 on targeted metabolites by LC-MS in H1299 cells.

**File Name: Supplementary Data 6**

Description: Examine the effects of MT-CO2 on ^13^C_5_-glutamine metabolic flux by LC-MS in H1299 cells.
